# Reproducible production and image-based quality evaluation of retinal pigment epithelium sheets from human induced pluripotent stem cells

**DOI:** 10.1038/s41598-020-70979-y

**Published:** 2020-09-01

**Authors:** Ke Ye, Yuto Takemoto, Arisa Ito, Masanari Onda, Nao Morimoto, Michiko Mandai, Masayo Takahashi, Ryuji Kato, Fumitaka Osakada

**Affiliations:** 1 Laboratory of Cellular Pharmacology, Graduate School of Pharmaceutical Sciences, Nagoya University, Nagoya, 464-8601 Japan; 2 Laboratory of Cell and Molecular Bioengineering, Graduate School of Pharmaceutical Sciences, Nagoya University, Nagoya, 464-8601 Japan; 3 Laboratory of Neural Information Processing, Institute for Advanced Research, Nagoya University, Nagoya, 464-8601 Japan; 4 Laboratory for Retinal Regeneration, RIKEN Center for Biosystems Dynamics Research, Kobe, 650-0047 Japan; 5 Department of Opthalmology, Kobe City Eye Hospital, Kobe, 650-0047 Japan; 6 Vison Care Inc., Kobe, 650-0047 Japan; 7 Institute of Nano-Life-Systems, Institutes of Innovation for Future Society, Nagoya University, Nagoya, 464-8601 Japan; 8 PRESTO/CREST, Japan Science and Technology Agency, Saitama, 332-0012 Japan

**Keywords:** Pluripotent stem cells, Regeneration, Stem-cell differentiation, Regenerative medicine, Stem-cell biotechnology, Tissue engineering, Therapeutics, Eye diseases, Pharmaceutics, Pharmacology

## Abstract

Transplantation of retinal pigment epithelial (RPE) sheets derived from human induced pluripotent cells (hiPSC) is a promising cell therapy for RPE degeneration, such as in age-related macular degeneration. Current RPE replacement therapies, however, face major challenges. They require a tedious manual process of selecting differentiated RPE from hiPSC-derived cells, and despite wide variation in quality of RPE sheets, there exists no efficient process for distinguishing functional RPE sheets from those unsuitable for transplantation. To overcome these issues, we developed methods for the generation of RPE sheets from hiPSC, and image-based evaluation. We found that stepwise treatment with six signaling pathway inhibitors along with nicotinamide increased RPE differentiation efficiency (RPE6iN), enabling the RPE sheet generation at high purity without manual selection. Machine learning models were developed based on cellular morphological features of F-actin-labeled RPE images for predicting transepithelial electrical resistance values, an indicator of RPE sheet function. Our model was effective at identifying low-quality RPE sheets for elimination, even when using label-free images. The RPE6iN-based RPE sheet generation combined with the non-destructive image-based prediction offers a comprehensive new solution for the large-scale production of pure RPE sheets with lot-to-lot variations and should facilitate the further development of RPE replacement therapies.

## Introduction

Human pluripotent stem cells (hPSC) [human embryonic stem cells (hESC) and human induced pluripotent stem cells (hiPSC)] are valuable sources for regenerative therapies because of their capacity for self-renewal and pluripotency^1^. Retinal regeneration using hPSC is a new promising treatment at the cutting edge of stem cell-based regeneration therapies. Retinal pigment epithelium (RPE) cells play important roles in vision and retinal function maintenance. Transplantation of RPE suspension or RPE sheets derived from hPSC for age-related macular degeneration (AMD) and Stargardt disease patients is considered safe and potentially effective^2–5^. However, this treatment faces challenges regarding the purity and quality control of RPE products for transplantation therapy. The properties of cellular products are totally different from those of chemical drugs that are well-established in the synthesis and quality control of drug development. For advancements in the industrialization of cell therapy, new approaches are needed that can solve the problems in large-scale production of cellular products with lot-to-lot variations.

To produce RPE cells from hPSC with high efficiency, many groups (including ours) have developed various protocols using only growth factor proteins or combinations with small molecules to recapitulate retinal development^6–12^. Small-molecule-based differentiation protocols have been developed for clinical application, but their efficiency and purity remain low^13,14^. Because only pure RPE can be used in transplantation, further purification of induced RPE is necessary, which is commonly achieved via manual selection by observing pigmentation of RPE under a microscope^4^. These procedures, however, need experienced operators and are not suitable for large-scale RPE production. Thus, effective methods for obtaining pure RPE sheets on large scales need to be developed for progress in regenerative medicine.

The other issue is the quality control of hPSC-derived cell products for transplantation therapy. The quality of cell products varies between lots. Lot-to-lot variation of cell products stems from both its nature of living creature cells and manual operation by humans^15,16^. It will be essential to determine the quality of every cell product before transplantation. Currently, the quality of cell products is determined by conventional methods such as DNA/RNA-based assays and immunolabeling, which destroy cell products themselves and are not suitable for transplantation therapy. ELISA and transepithelial resistance (TER) measurement are standard methods to evaluate the function of RPE sheets^17^, but they are low-throughput and time-consuming. Microscopic image-based analyses have been expected to assist in the evaluation of cell products^18^. Functional prediction models based on microscopic observation of the degree of pigmentation in RPE cells have been reported^19,20^. The morphological features of cells contain additional information about the cellular status and have been conventionally used in the daily monitoring of cells. While we have previously established machine learning models to estimate the quality of other cell products^21,22^, a non-destructive and quantitative prediction method based on morphological features of RPE cells has been lacking.

Here we developed a protocol for generating pure RPE sheets from hiPSC with high efficiency. Stepwise treatment of hiPSC with six signaling pathway inhibitors along with nicotinamide efficiently indued RPE differentiation (RPE6iN). Replating RPE6iN-induced RPE cells onto transwell produced mature RPE sheets at high purity without manual selection. We found lot-to-lot variations of barrier function between mature RPE sheets, consistent with general issues reported in previous studies^19,23^. To support the robust RPE sheet production ability, we developed a machine learning-based prediction model to predict failure samples only from their non-labeled cellular morphologies in microscopic images. This differentiation method, combined with the machine learning-based evaluation model, should be useful for efficient production and quality management of RPE sheets for regenerative cell therapies.

## Results

### Increases in purity of hiPSC-derived RPE cells by nicotinamide

Three cell lines of hiPSC, clones 1383D6, 1383D2, and A18945, were maintained under a feeder-free and xeno-free condition^24^. These hiPSC colonies displayed typical features, with tightly packed cells and large nuclei (Fig. S1A,B: 1383D6). To determine whether the hiPSC were pluripotent, immunostaining for OCT4, a marker for pluripotent stem cells, confirmed that every hiPSC colony expressed OCT4 (Fig. S1C: 1383D6). The lectin rBC2LCN binds to unique glycans on the cell surface of undifferentiated hESC and hiPSC^25^, and staining with rBC2LCN-rhodamine also revealed that all colonies were positive for rBC2LCN (Fig. S1D: 1383D6). Two lines 1383D2 and A18945 also expressed OCT4 (Fig. S1E,F). These results indicate that the hiPSC used in this research maintained a pluripotent state under our feeder-free condition.

**Figure 1:**
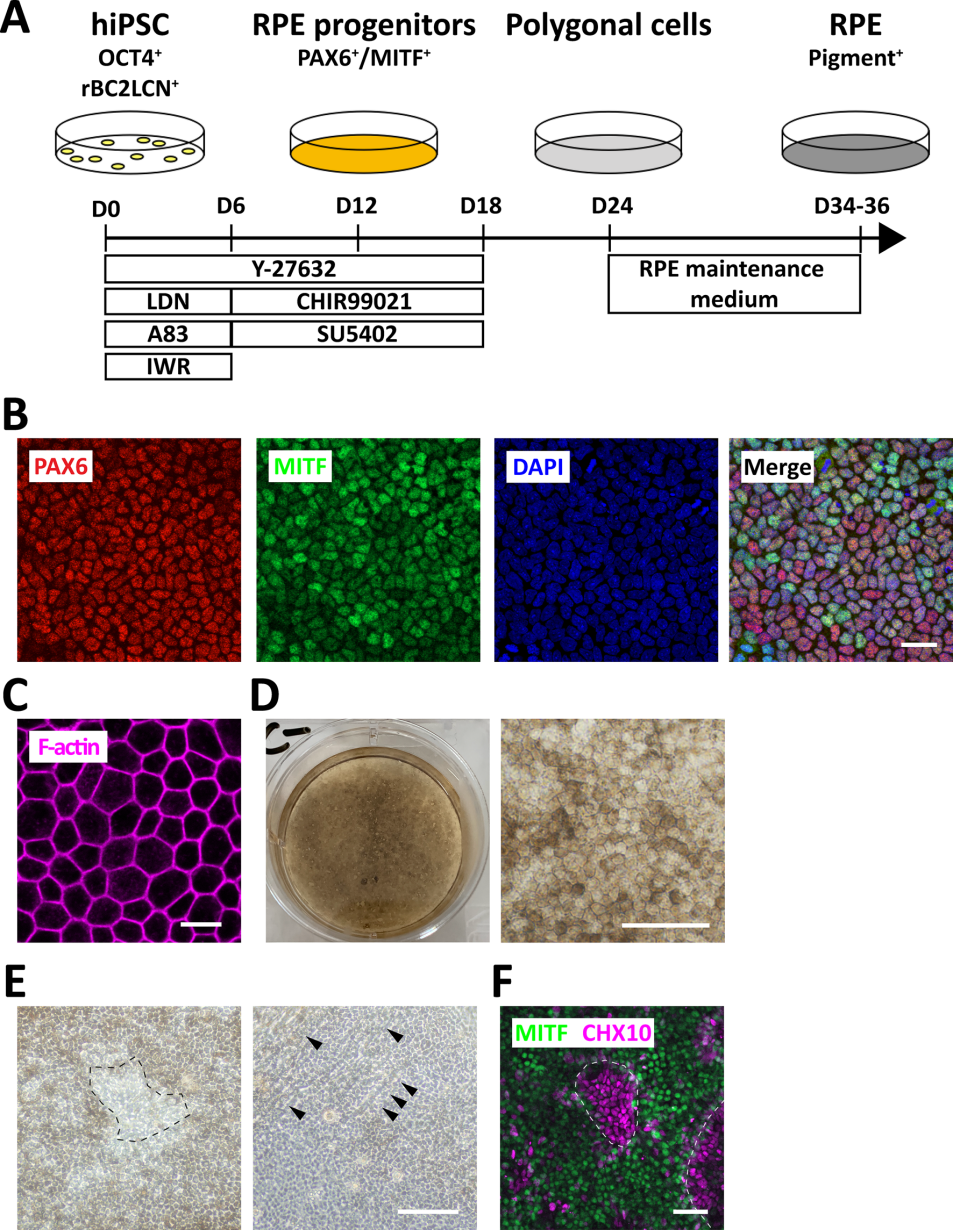
Small-molecule-based differentiation of RPE from hiPSC. (**A**) Timetable for stepwise treatment for RPE differentiation from hiPSC. Y27632 (10 µM), LDN (LDN193189, 100 nM), A83 (A83-01, 500 nM), IWR (IWR-1-*endo*, 1 µM), CHIR99021 (3 µM), and SU5402 (2 µM) were added to the culture medium for indicated periods. (**B**) Generation of RPE progenitors from hiPSC. Differentiated cells on day 12 were processed for immunostaining for PAX6 and MITF. (**C**) Generation of polygonal cells from hiPSC. Differentiated cells on day 32 were processed for phalloidin staining. (**D**) Generation of RPE from hiPSC. Macroscopic photographic images (left) and phase-contrast images (right) of differentiated cells on day 35. Note that most cells are pigmented and polygonal. (**E**) Co-induction of non-RPE cells from hiPSC. Dotted lines mark the non-pigmented cells (left). Arrow heads indicate neural process-like structure of the differentiated cells other than RPE on day 35 (right). (**F**) Minor populations of neural retinal progenitors. Representative immunostaining for MITF (a marker for RPE progenitors) and CHX10 (a marker for neural retinal progenitors) on day 35. Dotted lines mark the CHX10-positive cells. Scale bars: 20 μm (**B**,**C**), 50 μm (**D**,**F**), and 100 μm (**E**).

To induce RPE from hiPSC, we aimed to induce ectodermal cells, retinal progenitors, and RPE along the stages of the developmental process in a stepwise manner (Fig. 1A)^26,27^. We optimized the dual-Smad inhibition method to efficiently induce the ectoderm from hPSC^28^. The BMP signaling inhibitor LDN193189 (LDN, 100 nM) and the TGF-β signaling inhibitor A83-01 (A83, 500 nM) were added to the medium during the period from day 0 to day 6 (Fig. 1A). IWR-1-*endo* (IWR, 1 μM), a Wnt/β-catenin signal inhibitor, was simultaneously added during the period from day 0 to day 6 to promote retinal differentiation. Cells were treated with the ROCK inhibitor Y-27632 (10 μM) until day 18 to inhibit cell death^29^. The induced cells were subsequently treated with the GSK3β inhibitor CHIR99021 (3 μM) and the bFGF receptor inhibitor SU5402 (2 μM) (Fig. 1A) because Wnt signaling activation promotes RPE differentiation^9,30^ and blockage of FGF signaling inhibits neural retina differentiation^9,31^. To determine whether the hiPSC differentiated into RPE lineages, we performed immunostaining for PAX6, a marker for the inner and outer layers of the optic vesicle and the optic cup^32^, and MITF, a marker for the outer layer of the optic vesicle and the optic cup^33^. MITF and PAX6 double-positive cells were observed on day 12 (Fig. 1B), indicating that hiPSC differentiated into RPE progenitors under our differentiation condition. To induce pigmented RPE, we changed the culture medium to the RPE maintenance medium from day 24 when induced cells adopted a polygonal morphology with a cobblestone appearance. F-actin staining with phalloidin-Rhodamine visualized the formation of polygonal actin bundles (Fig. 1C). The polygonal cells accumulated pigmentation on day 35 (Fig. 1D). However, some non-pigmented cells with neural process-like structures were also observed on day 35 (Fig. 1E). Since both neural retina progenitors and RPE progenitors are derived from common progenitors, it is possible that the contaminated non-RPE cells were neural retina progenitors^9^. We examined whether the contaminated non-RPE cells were neural retina progenitors by immunostaining for CHX10, a marker for neural retina progenitors^34^, and MITF. A small number of cells were CHX10-positive and MITF-negative on day 35 (Fig. 1F), suggesting that the non-RPE cells were neural retina progenitors that were induced along with RPE cells from hiPSC. These results indicate that the stepwise treatment with the small molecules effectively induced RPE progenitors and RPE from hiPSC, with a minority of neural retina progenitors.

**Figure 2:**
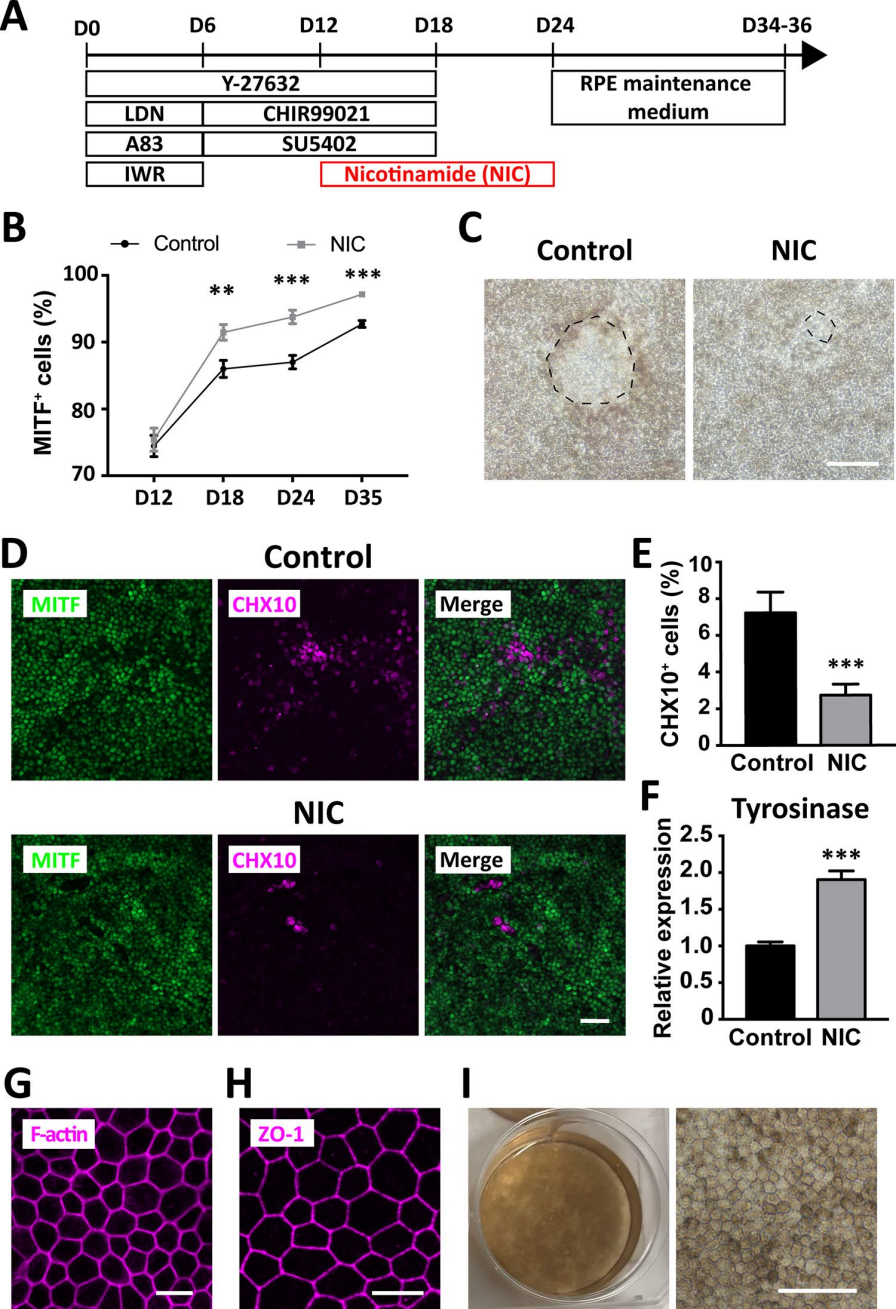
Efficient generation of pure RPE by RPE6iN method. (**A**) Timetable for the new differentiation method RPE6iN. Dissociated hiPSC were cultured on iMatrix 511-coated dishes in the presence of Y27632 (10 µM), LDN (LDN193189, 100 nM), A83 (A83-01, 500 nM), IWR (IWR-1-endo, 1 µM), CHIR99021 (3 µM), and SU5402 (2 µM) for indicated periods. NIC (Nicotinamide, 10 mM) was added into the culture medium from day 12 to day 24. (**B**) Promotion of RPE differentiation by NIC treatment. The percentage of MITF-positive cells were determined by immunostaining for MITF on days 12, 18, 24, and 35. **P < 0.01, ***P < 0.001, compared with control. (**C**) Phase-contrast images of induced cells with or without NIC treatment on day 35. Dotted lines mark the non-pigmented cells. Note that NIC treatment decreased the non-pigmented area. (**D**) Representative photomicrographs showing RPE6iN-treated hiPSC. HiPSC were differentiated in the presence or absence of NIC, fixed on day 35, and then subjected to immunostaining for MITF and CHX10. (**E**) The decrease in the percentage of CHX10-positive cells by NIC treatment on day 35. ***P < 0.001, compared with control. (**F**) Promotion of RPE maturation by NIC treatment. Gene expression of tyrosinase was quantified by RT-qPCR on day 24. ***P < 0.001, compared with control. (**G**) Generation of polygonal cells from RPE6iN-treated hiPSC. Differentiated cells were processed for phalloidin staining on day 31. (**H**) Formation of tight junctions in RPE6iN-treated hiPSC. RPE6iN-indued cells were immunostained for the tight junction marker ZO-1 on day 35. (**I**) Macroscopic photographic images and phase-contrast images of RPE6iN-indued pigmented cells on day 35 in culture. RPE6iN-treated cells were cultured in RPE maintenance medium. Scale bars: 20 μm (**G**,**H**), 50 μm (**D**,**I**), and 100 μm (**C**).

To induce pure RPE from hiPSC, we next sought to eliminate the neural retinal progenitors in the above protocol. Nicotinamide (NIC) promotes RPE differentiation^7,8,12,14^; therefore, we added NIC (10 mM) from day 12 to examine its effect on RPE differentiation (Fig. 2A). Immunocytochemistry for the RPE progenitor maker MITF revealed that NIC treatment significantly increased MITF-positive cells on day 18, 24 (Fig. S2A), and 35 (Fig. 2D), and the percentage of MITF-positive cells under NIC treatment on day 35 was 97.2 ± 0.27% (Fig. 2B). Notably, we observed more cells with polygonal morphology and fewer non-pigmented cells on day 35 under NIC treatment (Fig. 2C). NIC treatment from day 12 to day 24 significantly decreased the number of cells positive for CHX10 on day 35 (Fig. 2D,E). We also found that NIC treatment promoted the transition from PAX6 and MITF double-positive cells to MITF-positive and PAX6-negative cells (Fig. S2B–E), suggesting that NIC promoted the RPE differentiation. qPCR analysis demonstrated the up-regulation of tyrosinase, an enzyme responsible for pigmentation in RPE, on day 24 with NIC treatment (Fig. 2F). These results indicate that NIC treatment induces high-purity RPE cells by promoting RPE differentiation and suppressing neural retinal differentiation.

To obtain pigmented RPE cells, we further cultured the hiPSC-derived cells in RPE maintenance medium for the promotion of RPE pigmentation^14^. F-actin staining revealed that pigmented cells formed polygonal actin bundles (Fig. 2G). The induced cells expressed ZO-1, a tight junction marker (Fig. 2H). The majority of the induced cells accumulated more pigmentation on day 35 (Fig. 2I). These results indicate that our differentiation protocol produced pigmented RPE from hiPSC. We referred the RPE differentiation method by stepwise treatment of dissociated hiPSC with 6 signaling pathway inhibitors RHOi, BMPi, TGFi, WNTi, FGFi, and GSK3βi along with NIC as “RPE6iN” hereafter.

To determine the stability of the RPE6iN method, we assessed the reproducibility of this method with three independent sets of experiments. RPE6iN-treated hiPSC consistently induced MITF-positive RPE progenitors and pigmented cells in every experimental trial (Fig. S3A,B). These results suggest that RPE6iN method consistently induced RPE from hiPSC with little variability. We next examined whether our RPE6iN method that has been developed using the clone 1383D6 can induce RPE differentiation in other lines of hiPSC. We used an additional two independent hiPSC lines (clones 1383D2 and A18945) to test the differentiation efficiency by the RPE6iN method. The three lines provided similar results in RPE differentiation; 82% of cells were positive for MITF for clone 1383D2, 76% for clone A18945, and 75% for clone 1383D6 on day 12 (Fig. S3C,D). The RPE differentiation efficiency was comparable among three independent lines. We conclude that RPE6iN method affords high yields of pure RPE cells with high reproducibility and low variability across multiple hiPSC lines.

### Generation of pure functional RPE sheets

**Figure 3:**
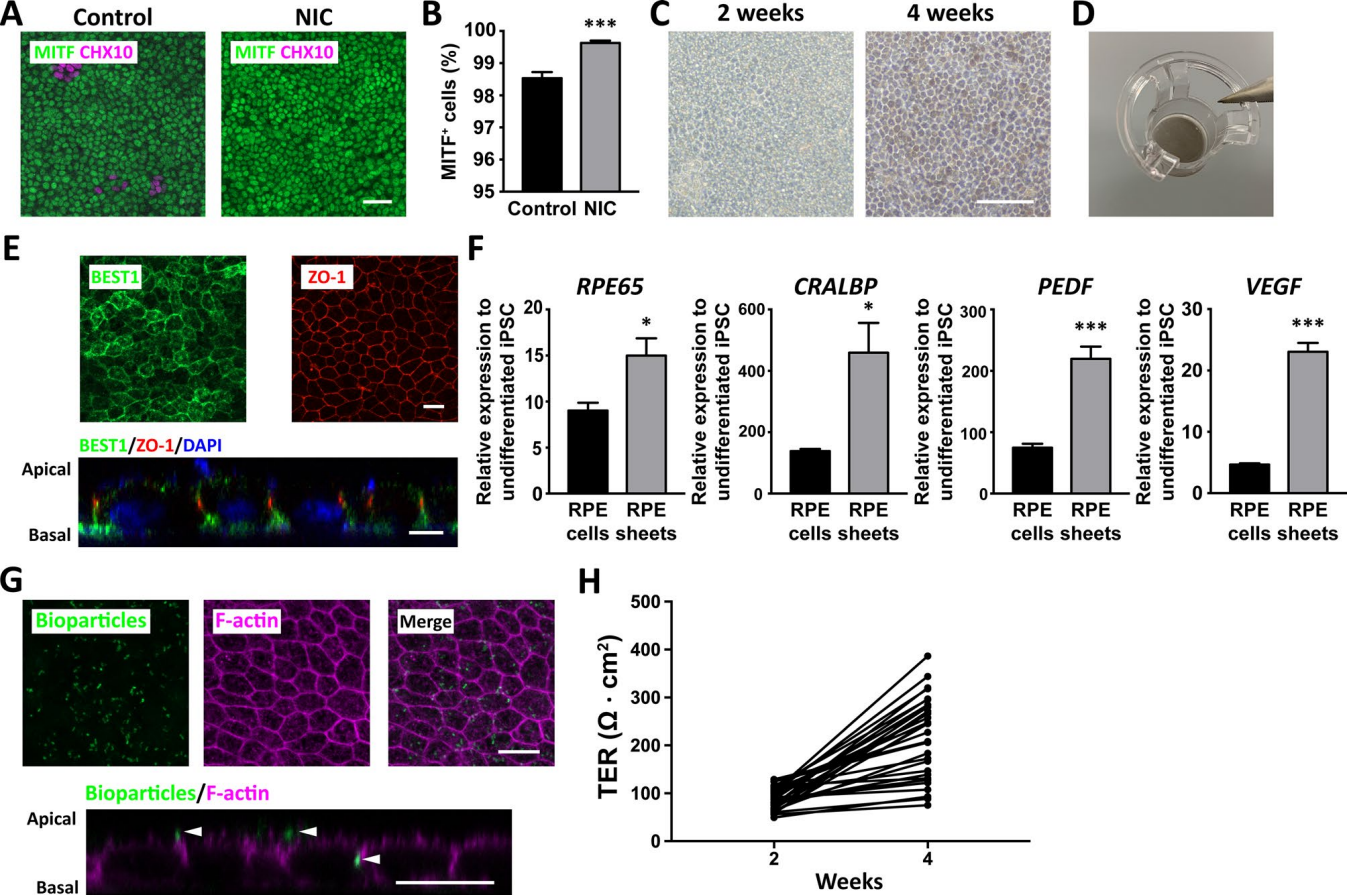
Generation of mature functional hiPSC-derived RPE sheets. (**A**) Representative photomicrographs showing expressions of MITF and CHX10 in RPE6iN-treated cells. HiPSC were differentiated in the presence or absence of NIC and replated for further culture for seven days. (**B**) The percentage of MITF-positive cells after NIC treatment. ***P < 0.001, compared to control. HiPSC were differentiated using NIC treatment, replated for expanded culture, and subjected to immunocytochemistry. (**C**) Sheet formation of RPE6iN-treated cells. HiPSC-RPE sheets were maintained for 2 and 4 weeks after seeding on the transwell. (**D**) Macroscopic photographs of hiPSC-RPE sheets after 4-week culture. (**E**) Maturation of hiPSC-RPE sheets. Representative x–y section and x–z section images showed that RPE sheets expressed both ZO-1, a tight junction marker located subapically, and BEST1, a basolateral marker of the mature RPE. RPE6iN-induced RPE cells were cultured on the transwell for 4 weeks after seeding and then processed for immunostaining. (**F**) Maturation of hiPSC-RPE sheets. Gene expression of *RPE65*, *CRALBP*, *PEDF*, and *VEGF* in RPE6iN-induced RPE cells (differentiation day 24) and RPE sheets relative to undifferentiated hiPSC was quantified using RT-qPCR. *P < 0.05, ***P < 0.001, compared to RPE cells. (**G**) Phagocytosis ability of hiPSC-RPE sheets. Representative x–y section (upper panel) and x–z section images (lower panel) of hiPSC-RPE sheets cultured with bioparticles at 37 °C. pHrodo-Green-conjugated bioparticles (green) become fluorescent at low pH, indicating the phagocytosis of bioparticles. F-actin (magenta) was labeled by phalloidin staining. (**H**) Evaluation of the barrier function of hiPSC-RPE sheets. TER values of hiPSC-RPE sheets on the transwell were measured 2 and 4 weeks after seeding on the transwell. Scale bars: 20 μm (**A**,**E**,**G**) and 100 μm (**C**).

Although transplantation of human ESC-derived RPE suspensions has been safely performed for AMD patients in clinical trials^5^, it is difficult for RPE in suspension to self-organize into a functional monolayer under the neural retina of patients, limiting the long-term survival and efficacy of the transplanted RPE^35^. Alternatively, producing and transplanting functional RPE sheets can improve the survival prospects of transplanted RPE^13,23,36^. Thus, we sought to produce hiPSC-derived RPE sheets that should be useful for such applications. We first assessed the purity of RPE6iN-induced cells after passage for sheet generation. More MITF-positive cells (> 99%) and less CHX10-positive cells (< 0.5%) were observed under NIC treatment after one-time passage of the induced RPE (Fig. 3A). NIC treatment significantly increased the MITF-positive cells (Fig. 3B). These results suggest that the high purity of mature RPE sheets can be produced by one bulk passage of the RPE6iN-induced RPE cells without any manual selection. We seeded the RPE6iN-induced RPE cells onto the transwell, which allows the RPE to grow into mature epithelial sheets^37^. Pigmentation of RPE cells increased four weeks after seeding onto the transwell in a culture-period-dependent manner (Fig. 3C,D). The RPE exhibits polarized epithelial structure along the apical-basal axis, a typical feature of the mature RPE. To examine apico-basal polarity and maturation of the RPE sheets, we assessed the expression of ZO-1, a tight junction protein located on the apical zone, and Bestrophin-1 (BEST1), a channel protein located on the basolateral plasma membrane of the RPE. ZO-1 was expressed in the apical zone of the RPE sheets, while BEST1 was localized at the basolateral side (Fig. 3E). These results indicate that the hiPSC-RPE sheets were mature and polarized in an apical-basal orientation, in line with the previous studies^2,38^. We next examined the expression of genes responsible for RPE maturation and polarization. qPCR analysis demonstrated that RPE6iN-induced cells expressed typical mature RPE makers: RPE65, an enzyme associated with the retinoid cycle; CRALBP, a retinoid-binding protein involved in the retinoid cycle; VEGF, a growth factor released from the basal side of the RPE; and PEDF, a growth factor released from the apical side of the RPE. hiPSC-RPE sheets showed higher expression levels for the four genes (Fig. 3F). Taken together, the sheets produced from RPE6iN-induced cells have typical features of the mature RPE, including pigmentation, polygonal and polarized morphology, and expression of genes and proteins responsible for tight junctions, polarized cytokine secretion, and visual cycles.

One of the essential functions of the polarized RPE monolayer is phagocytosis of the outer segment shed by photoreceptors^39^. Therefore, we evaluated the phagocytic capability of hiPSC-RPE sheets using pH-Rhodo-labeled bioparticles. Numerous fluorescent bioparticles were observed in the apical and cytoplasmic location of RPE sheets after exposure for 4 h at 37 °C (Fig. 3G). Conversely, under the negative control (4 °C), the RPE sheets showed few fluorescence signals (Fig. S4)^5^. These results indicate that the hiPSC-RPE sheets were capable of phagocytosis. Barrier function is another important property of highly polarized RPE sheets^37^. TER measurement is commonly used for measuring the barrier function of RPE sheets with high sensitivity and reliability^20,37^. To evaluate the barrier function of hiPSC-RPE sheets, we measured the TER values at different time points. The TER values of the hiPSC-RPE sheets increased during culture time (Fig. 3H). The TER values were different among RPE sheets, even under the same culture condition, and some RPE sheets did not reach high TER values (Fig. 3H), which is consistent with previous studies^19,23^. We conclude that functional RPE sheets were generated after further culture of the pure hiPSC-RPE cells on the transwell and that the quality of the hiPSC-RPE sheets varied among lots.

### Abnormal tight juntions, adherens junctions, and F-actin cytoskeleton in hiPSC-RPE sheets with low TER values

**Figure 4:**
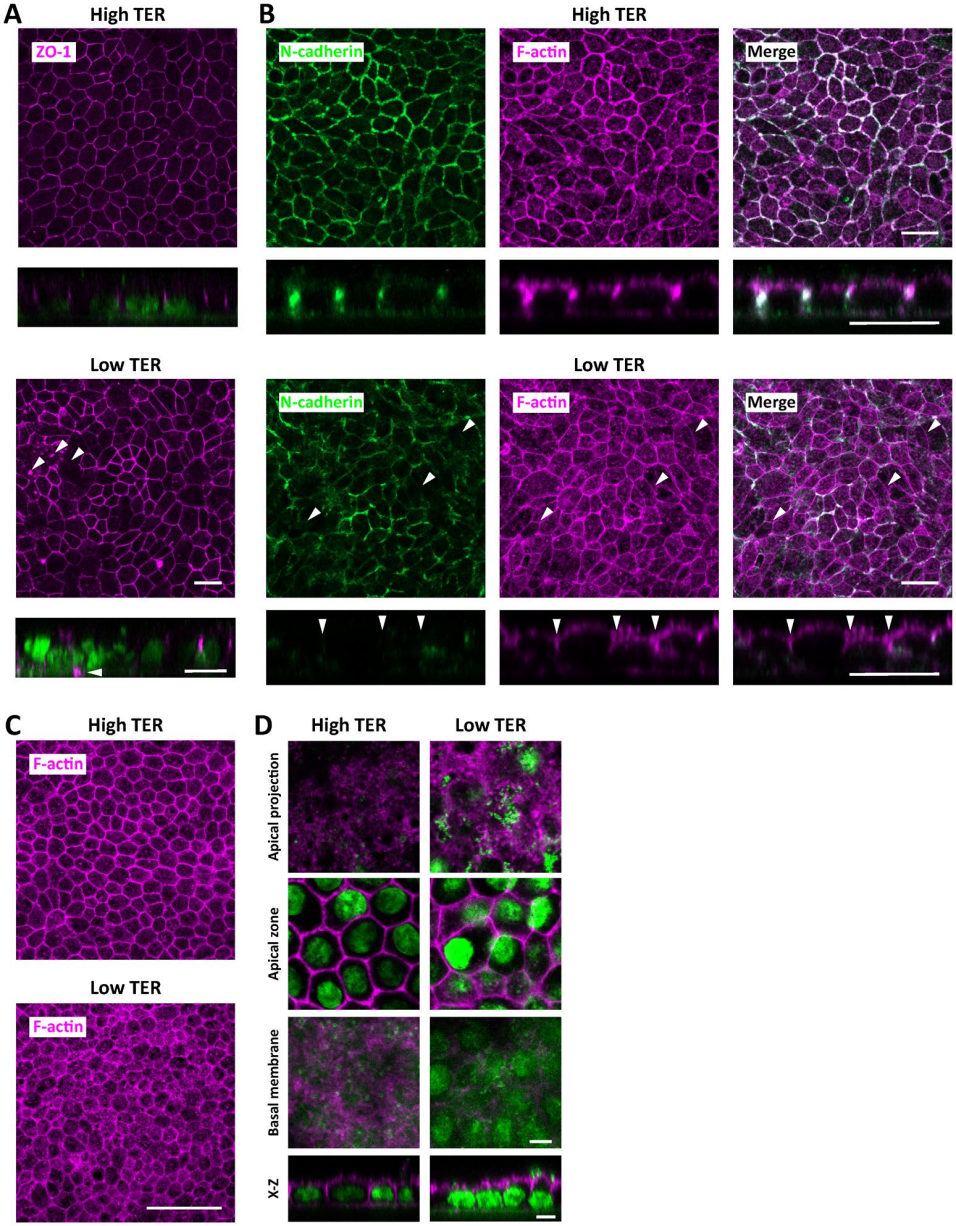
Abnormal cytoskeleton structure in hiPSC-RPE sheets with low TER values. (**A**) ZO-1 distribution of hiPSC-RPE sheets with high (375 Ω cm^2^) and low (113 Ω cm^2^) TER values in x–y confocal sections (upper panel) and x–z confocal sections (lower panel). The x–z confocal sections represent merged images for ZO-1 (magenta) and nuclei (green). REP6iN-treated hiPSC were cultured in transwells and subjected to TER measurement and immunostaining. Arrowheads indicate the mislocalization of ZO-1. (**B**) Representative photomicrographs of N-cadherin and F-actin staining of RPE sheets with high (259 Ω cm^2^) and low TER values (125 Ω cm^2^). Arrowheads indicate cells with low expression of N-cadherin. (**C**) F-actin distribution in hiPSC-RPE sheets. Representative phalloidin staining for F-actin in RPE sheets with high (386 Ω cm^2^) and low (93 Ω cm^2^) TER values. RPE6iN-induced RPE cells were cultured in transwells and subjected to TER measurement and phalloidin staining. (**D**) Subcellular localization of F-actin in hiPSC-RPE sheets. Representative images were obtained using identical imaging settings showing F-actin (magenta) and nuclei (green) in separate individual x–y confocal sections. Individual sections are shown for apical, medial, and basal aspects of cells and an x–z section of the same field of RPE sheets with high (386 Ω cm^2^) and low (93 Ω cm^2^) TER values. Scale bars: 10 μm (**D**), 20 μm (**A**,**B**), and 50 μm (**C**).

Given the variability of TER values between hiPSC-RPE sheets (Fig. 3H), we examined potential differences between RPE sheets with high TER values and those with low TER values. Many lines of evidence have demonstrated that both tight junctions and adherens junctions are essential for barrier function^40,41^. Localization of ZO-1 in tight junctions on the apical side of RPE cells was identified (Fig. S5A). We first assessed how ZO-1 was distributed within hiPSC-RPE sheets with low TER values. Since it has been reported that the TER value of a human RPE monolayer in vivo is 150 Ω cm^37,42^, we categorized hiPSC-RPE sheets with TER values > 250 Ω cm^2^ into a high-TER value group. HiPSC-RPE sheets with TER values < 150 Ω cm^2^ were categorized into a low-TER-value group. In RPE sheets with high TER values, ZO-1 was distributed in a polygonal morphology with a squamous appearance (Fig. 4A). However, ZO-1 in RPE sheets with low values lacked the typical squamous pattern, with patchy disappearance of ZO-1 expression. Moreover, ZO-1 was mislocalized to the basolateral side of RPE sheets with low TER values because of multiple cellular layers (Figs. 4A, S5C), whereas ZO-1 was distributed between cells on the apical side in RPE sheets with high TER values (Fig. 4A). We next focused on adherens junctions comprising cadherins and catenins^43^. Cadherins are required for the maintenance of the integrity of epithelial cells^44^, whereas catenins link to actin filaments. Three major cadherin proteins, N-, E-, and P-cadherins, are localized to the lateral membranes of RPE cells^45^. We found that N-cadherin co-localized with lateral F-actin in the intercellular membrane (Fig. S5B). To determine whether adherens junction proteins are also lost in malfunctioning RPE sheets, we examined distribution patterns of N-cadherin in the RPE sheets. In RPE sheets with high TER values, N-cadherin was localized to the lateral membrane of cells from the apical to the basal side (Fig. 4B). In RPE sheets with low TER values, N-cadherin expression was lost (Fig. 4B). These results suggest that both tight junctions and adherens junctions are disorganized in hiPSC-RPE sheets with low TER values.

Adherens junctions are linked to the organization of F-actin in cells^43^. Abnormalities in the actin cytoskeleton affect adherens junction formation, and disruption of microfilaments perturbs cell–cell adhesion^46–48^. Normal polarized RPE cells possess F-actin in the transcellular membrane microvilli, circumferential microfilament bundles, and basal infolding^49^. However, accumulating evidence has demonstrated irregular distribution of F-actin in aged human RPE and the RPE of AMD patients^50^. The cytoskeleton has also been shown to affect the distribution of apical surface area in epithelial sheets^51^. Thus, to examine the relationship of the cytoskeleton to RPE sheet quality, we determined how F-actin was distributed in RPE sheets. F-actin was localized in circumferential bundles in RPE sheets with high TER values and more diffusely in RPE sheets with low TER values (Fig. 4C). We further evaluated the subcellular localization of F-actin and discovered a loss of lateral and basal F-actin of some RPE cells in RPE sheets with low TER values, while F-actin was evenly localized on the apical, central, and basal sides in RPE sheets with high TER values (Figs. 4D, S5D). These results indicate that hiPSC-RPE sheets with low TER values lost F-actin on the lateral and basal sides. Thus, we conclude that RPE sheets with low TER lose tight junctions and adherens junctions and have irregular F-actin localization.

### Morphology-based prediction model for TER values of RPE sheets

For clinical application purposes, any RPE sheets with abnormal barrier function need to be detected and eliminated before sheet transplantation. Our findings suggest that the cellular morphology of RPE sheets with low TER values is different from those with high TER values because of differences in F-actin distribution. VEGF secretion is one of the key parameters for RPE function. However, the quantification of VEGF secretion varies between experiments because values that are obtained from ELISA for VEGF are affected by the number of RPE cells, the amount of culture medium, and time for accumulation of secreted VEGF. TER measurement is more reproducible and reliable than ELISA for VEGF. Thus, we hypothesized that a TER prediction method based on the morphology of RPE cells could identify abnormal RPE sheets in a non-invasive way.

**Figure 5:**
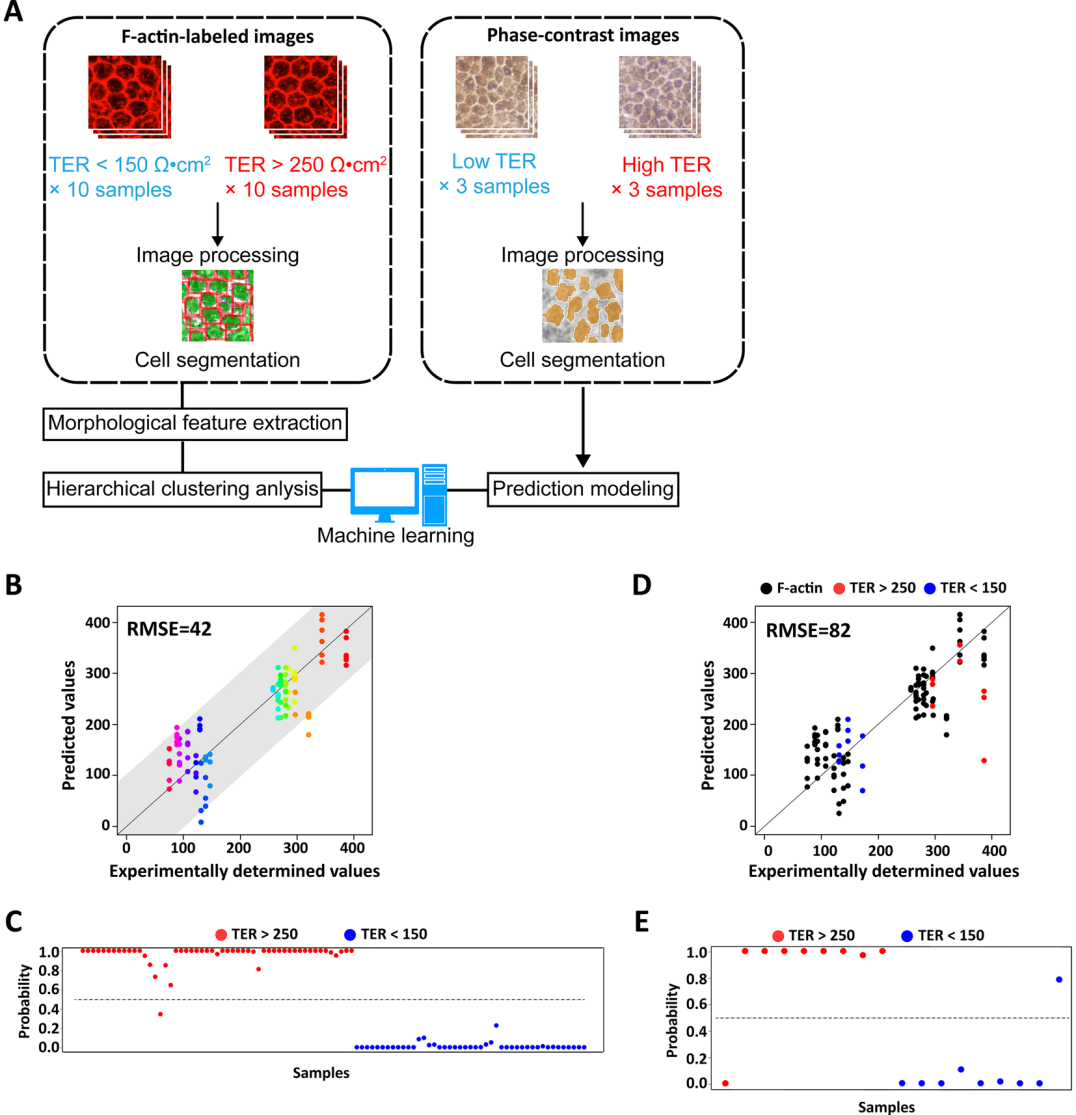
Morphology-based TER value prediction for hiPSC-derived RPE sheets. (**A**) Schematic illustration of image processing, feature extraction, hierarchical clustering, and prediction modeling. (**B**) Prediction modeling of hiPSC-RPE sheet function using F-actin labeled images. A plot shows performance of TER predictions from machine learning of the morphological features from F-actin-labeled images against the measured TER values. The black diagonal line represents a perfect prediction. RMSE: Root mean squared error. (**C**) Two-choice discrimination of hiPSC-RPE sheets using F-actin labeled images. A plot of TER discrimination of the morphological features from F-actin-labeled images against the test samples of F-actin-labeled images. Red plot represents the test samples in high TER values. Blue plot represents the test samples in low TER values. (**D**) Prediction of hiPSC-RPE sheet function using label-free, phase-contrast images. Plot shows performance of TER predictions from machine learning of the morphological features from F-actin labeled images against the measured TER values in phase-contrast images. Black plot represents the samples for TER values prediction model construction. Red plot represents the test samples in high TER values. Blue plot represents the test samples in low TER values. (**E**) Two-choice discrimination of hiPSC-RPE sheets using label-free, phase-contrast images. A plot of TER discrimination of the morphological features from F-actin labeled images against the test samples of phase-contrast images. Red plot represents the test samples with high TER values. Blue plot represents the test samples with low TER values.

We sought to develop a morphology-based prediction model using F-actin-labeled cells in hiPSC-RPE sheets. The workflow for dataset construction and morphology-based prediction is presented in Fig. 5A. Analysis of multiple morphological profiles (Fig. S6 and Table S2) with their experimentally determined TER values via clustering revealed that the morphological profiles included several varieties of sub-population types showing a different correlation than the major trend (Fig. S7A). These results imply biological heterogeneity of RPE sheets, which can be noise in the analysis. To construct a general morphological model to predict TER values, we thus cleansed the samples to include samples that showed a correlation to TER values for noise reduction and majority extraction (Fig. S7B). We picked up 30 samples with TER < 150 Ω cm^2^ as a low-TER value group and another 30 samples with TER > 250 Ω cm^2^ as a high-TER value group. Using these datasets of morphological profiles (16 morphological parameters) and TER values, we constructed two types of prediction models: (1) a TER-value prediction model and (2) a high vs. low TER discrimination model.

Using only the morphological profiles of RPE sheets, the TER-value prediction model predicted TER values with a low error rate (Fig. 5B). Both high-TER samples and low-TER samples showed a linear correlation with the morphological parameter combinations. The major weights assigned to morphological parameters in the model were “Average of length/width ratio” and “SD of compactness” for negative effects, and “Average of width” and “SD of length/width ratio” for positive effects (Table S3). These morphological profiles can be interpreted to indicate that the TER value is low when cells are disordered and heterogeneous in shape (i.e. with greater variation of long and short cells). The morphological explanation was also effective for the TER discrimination model (Fig. 5C). Using only the morphological parameters, the discrimination performance was high (accuracy: 99%, sensitivity: 98%, specificity: 100%). Regarding the weights in the discrimination model, “SD of compactness” showed the strongest negative effect, while “Average of width” had the strongest positive effect (Table S4). These weight combinations can be interpreted as describing the same phenotype as the TER-value prediction model.

Although the importance of morphology in predicting TER values was apparent, the model constructed only using labeled images cannot be applied for the non-invasive evaluation of RPE sheets during their culture process. To make our morphological model more practically applicable for use across various facilities, we sought to adapt the labeled-image-based prediction model to the morphological information of the non-labeled images. To achieve this, we picked 6 samples with phase-contrast microscopic images of RPE sheets and traced the clearly visible cellular borders in the images. Low-TER samples (150 ± 12 Ω cm^2^) and high-TER samples (342 ± 26 Ω cm^2^) were selected (Fig. 5A). With the ImageJ plugin Cell Magic Wand and minimal manual assistance, the additional cellular morphological analyses could be processed automatically, even from non-labeled images (Fig. S8). The cells measured by this process were converted into the same 16 explanatory parameter sets and then evaluated by the above prediction models. The TER-value prediction model performed well for most of the selected samples. Only one sample with high TER value was underestimated by the TER-value prediction model (Fig. 5D). When samples were analyzed by the TER discrimination model, the discrimination performance was practically effective (accuracy: 88%, sensitivity: 88%, specificity: 88%) (Fig. 5E). Thus, our morphology-based quality prediction model trained with labeled images could be readily adapted for the evaluation of non-labeled samples.

We next examined whether our prediction model could predict the TER values of RPE sheets that were generated from other hiPSC cell lines with other differentiation protocols across various facilities. Six hiPSC-RPE sheets were generated from different cell lines in clinical manufacturing facilities^4^. Three RPE sheets were derived from the iRTA-01 line of hiPSC, two were derived from the 253G1 line, and one was derived from the Ff-I01 line. Non-labeled images from the six hiPSC-RPE sheets were subjected to morphological analysis and our prediction model based on the 1383D6 line. Our model was able to predict TER values in all 6 of the hiPSC-RPE sheets (Fig. S9). The RMSE value for multiple hiPSC lines was 81, whereas that for the hiPSC line which were used for the model construction was 82. Prediction performance across different cell lines was comparable to that within the parental cell line. These results indicate that our morphology-based prediction method can be successfully applied to data from other facilities and RPE sheets. We conclude that a machine learning-based prediction model will increase the accuracy and efficiency of selecting hiPSC-RPE sheets with TER functionality and excluding failed hiPSC-RPE sheets manufactured for cell therapy.

## Discussion

Here we developed the differentiation method RPE6iN to efficiently induce differentiation of hiPSC into RPE cells, enabling the preparation of high-purity RPE cells and mature RPE sheets without special selection. We also constructed a non-invasive, machine learning-based TER prediction model from F-actin-labeled images to discriminate RPE sheets with low TER. This prediction model was also extended to be practically effective when used with non-labeled RPE images even across different cell lines and facilities. As detection and discrimination of every single cell product are necessary for cell manufacturing, our non-destructive, image-based prediction model will contribute to the discrimination of failure products during manufacturing. The efficient generation of pure RPE sheets from RPE6iN-treated hiPSC combined with the assistance of the morphology-based functional prediction system will be useful for large-scale production and quality control of RPE sheet products in industrialization and facilitate the development of cell therapies.

Several lines of evidence demonstrated the importance of lateral membrane in epithelial functions^51,52^. The hiPSC-RPE sheets with low TER values tended to lose lateral F-actin, consistent with the importance of lateral F-actin in the maintenance of the epithelial architecture. We found piled-up cells in RPE sheets with low TER values. A potential reason for why RPE sheets lost their epithelial architecture is that the RPE cells maintained the proliferation potential even after their growth reached confluency. Unlike the human fetal RPE, which cannot be passaged multiple times, hPSC-RPE cells are capable of keeping the epithelial morphology even after 13 times passage^53^, suggesting that hPSC-RPE has the developmental potential to proliferate more. However, the proliferation potential might make the hPSC-RPE difficult to mature and polarize when functional RPE sheets are required for transplantation therapy. Blocking cell cycles has been shown to promote maturation and polarization of RPE sheets, ciliogenesis, and TER values^54^, suggesting that controlling cell proliferation is important for RPE maturation. Collectively, controlling the cell proliferation and contractile balance of the apical-lateral axis may improve the production of high-quality RPE sheets. This might help in the development of a suitable culture condition for the functional RPE sheet generation.

The establishment of stable and reproducible quality control of differentiated cells is an urgent task for the clinical application of hPSC. Variabilities in hiPSC^55^ and hiPSC-derived products such as cardiomyocytes^56^ have been reported due to manual operation for a long period of cell culture. The quality of generated RPE sheets also can vary among different hiPSC lines^13^ and among different protocols in the same hiPSC line^57^. Consistent with previous reports^19,23^, we also found that TER values varied between RPE sheets even using the same hiPSC cell line and the same culture protocol. For industrialization of cellular products for regenerative medicine, it must be ensured that every product is fully functional. However, it has been almost impossible to evaluate the function of every RPE sheet. Thus, developing methods that discriminate between qualified and rejected RPE sheets is crucial for regenerative medicine.

Morphological features of live cells have been used for identifying the variable qualities in other hiPSC-derived cells such as hiPSC-derived cardiomyocytes^56^. The pigmented appearance of RPE was used to predict tissue maturity and functionality in a non-invasive manner^19,20^. However, detecting the variation of single RPE sheets remains inefficient, and little is known about morphological features in stem cell-derived RPE sheets^58^. Herein, the morphological rules extracted by the computational model trained only with labeled images can be adapted to the morphological features in the non-labeled status of RPE sheets even across hiPSC lines in different facilities. Currently, the prediction model approach for testing cell quality in cell manufacturing facilities for cell therapy faces two practical difficulties. One is the limitation of sample size for model training. It is difficult to accumulate thousands of images in cell manufacturing facilities before clinical treatment. However, the use of quality prediction models can enhance the stability of cell-based products even with a small sample size. From this aspect, our method provides insight into establishing an RPE sheet evaluation process using a half-manual but simple method that can be introduced without complete parameter studies using deep learning models. Our simple prediction model is more practical and flexible than deep learning models. Another critical issue is the reliability of prediction models. Because each cell manufacturer needs to show its own evidence regarding the manufacturing process, safety, and efficacy of its cellular products to the regulatory stakeholders, the evidence is required to be explained by its own data obtained with its protocol. Therefore, it is impossible for cell manufactures to “borrow” or “buy” data obtained by other facilities for their evidence because protocols are different between manufacturers. In other words, cell manufacturing facilities cannot use prediction models trained by data from different facilities with maximum reliability. We propose that each cell manufacturing facility that produces products for regenerative medicine should establish its own facility-specific prediction models using in-house data, rather than general models. This study supports the concept that any facilities can construct their prediction models based on their own system, including their cell lines, differentiation protocols, and microscope systems, because the construction of such a simple prediction model does not need a large number of labeled images.

Banking cells will be essential for regenerative medicine. Banking hiPSC with homozygous HLA at three loci and HLA-modified universal hiPSC may open a new avenue in regenerative medicine^59–61^. Banking differentiated cells may also be practical in regenerative medicine because differentiation of hiPSC to RPE cells and subsequent generation of RPE sheets take time and require various skills, personnel, and quality control testing in each facility. However, making frozen stocks of RPE sheets has not been possible since thawing frozen RPE sheets significantly induces cell death and collapses the sheet structure. Conversely, cryopreservation of hiPSC-RPE cells is possible (Fig. S10). Banking cells at the RPE stage can reduce the time required for RPE sheet production. Importantly, transplantation of RPE cell suspensions is also an effective strategy for RPE replacement therapy depending on the type of patient. Thus, hiPSC-RPE banks may facilitate transplantation strategies using RPE cell suspensions and RPE sheets for retinal regenerative medicine.

## Materials and methods

All experiments in this study were conducted with the approval of Nagoya University and in accordance with the Guidelines of Nagoya University.

### Culture of human iPSC

The human iPSC lines (clones: 1383D2 and 1383D6) were generated from a non-HLA-homozygous donor under feeder-free conditions and provided by Drs. Masato Nakagawa and Shinya Yamanaka of the Center for iPS Cell Research, Kyoto University^24^. The experimental protocols dealing with human subjects were approved by the institutional review board at Ethics Committee of Graduate School and Faculty of Medicine, Kyoto University. Written informed consent was provided by each donor. The clones 1383D2 and 1383D6 were generated from peripheral blood cells by five plasmids (pCE-hOCT3/4, pCE-hSK, pCE-hUL, pCE-mp53DD and pCXB-EBNA1) for induction of pluripotency. The human iPSC line (clone: A18945) was purchased from Thermo Fischer. The clone A18945 was generated from CD34+ human cord blood cells by three episomal vectors of seven factors SOX2, OCT4, KLF4, MYC, NANOG, LIN28, and SV40L T antigen. The human iPSC were maintained on iMatrix 511 (Nippi Inc., Tokyo, Japan)-coated culture dishes in StemFit AK02N (Ajinomoto, Tokyo, Japan)^24^. For passaging, when hiPSC grew almost to confluency, hiPSC were treated with accutase (Nacalai Tesque, Kyoto, Japan), dissociated into single cells, and replated at 5.0 × 10^3^ cells/cm^2^ onto iMatrix 511-coated dishes in AK02N in the presence of 10 μM Y-27632 (Wako, Osaka, Japan) for 24 h. The medium was changed to a fresh one without Y-27632 on the next day of plating and thereafter every 2 days. hiPSC were maintained in a humidified atmosphere of 5% CO_2_ and 95% air at 37 °C. hiPSC at passage 6 to 13 were used for the present study to guarantee pluripotency. Data from the clone 1383D6 were presented in “Results” and Figures, unless otherwise indicated.

### RPE differentiation and RPE sheet production

For differentiation, undifferentiated hiPSC were treated with accutase for 5 min, dissociated into single cells, and plated onto iMatrix 511-coated 6-well culture plates (Thermo Fisher Scientific, Waltham, MA) at 3.0 × 10^4^ cells/well. For RPE induction, cells were treated with 100 nM LDN193189 (Sigma, St. Louis, MO), 500 nM A-83-01 (Wako), 1 μM IWR-1-*endo* (Wako), and 10 μM Y-27632 were added to IMDM/Ham’s F12 (1:1, both from Sigma) supplemented with 10% KnockOut Serum Replacement (Thermo Fisher Scientific), 0.5 mM Monothioglycerol Solution (Wako), 1% Chemically Defined Lipid Concentrate (Wako), and 2 mM l-glutamine (Wako) for the initial 6 days, and then with 3 μM CHIR99021 (Wako), 2 μM SU5402 (Wako), and 10 μM Y-27632 in IMDM/F12 for another 12 days. From day 18, the medium was changed to DMEM/F12 (Sigma) supplemented with 10% KnockOut Serum Replacement, 1% N2 Supplement (Wako), and 2 mM l-glutamine. In some experiments, 10 mM nicotinamide (Wako) was added from day 12 to day 24. For further maturation, hiPSC-RPE were cultured in RPE maintenance medium (67% high glucose DMEM (Wako), 29% Ham’s F12, 2% B27 supplement minus vitamin A (Thermo Fisher Scientific), 2 mM l-glutamate, 100 U/mL Penicillin and 100 μg/mL Streptomycin. The culture medium was changed with a fresh one every day.

For RPE sheet generation, the hiPSC-RPE were treated with 0.25% Trypsin–EDTA (Wako) for 10 min and dissociated into single cells by pipetting. The cells were filtered by passing through a 35-μm cell strainer (Corning Inc., Corning, NY) and plated onto iMatrix 511-coated 12-well transwell insert (Corning) for functional analysis or iMatrix 511-coated 6-well culture plates for further proliferation. For the functional analysis, hiPSC-RPE were cultured in RPE growing medium containing Ham’s F12 with 10% fetal bovine serum (Thermo Fisher Scientific), 2 mM l-glutamate, 100 U/mL Penicillin and 100 μg/mL Streptomycin. After 14 days, the hiPSC-RPE growing medium was replaced with RPE maintenance medium containing 10 ng/mL basic fibroblast growth factor (Wako), and 0.5 μM SB431542 (Wako)^13^. All the cells were used until passage 3.

### Quantitative real-time polymerase chain reaction

Total RNAs of hiPSC and differentiated cells were extracted using Tissue Total RNA Mini Kit (Favorgen Biotech Corp., Taiwan), and then reverse-transcribed with PrimeScript RT Master Mix (TaKaRa, Shiga, Japan) according to the instructions of the manufacturer. Quantitative real-time PCR was performed with TB Green Fast qPCR Mix (TaKaRa) on LightCycler 96 system (Roche, Indianapolis, IN). Expression levels were normalized to those of GAPDH. The primers used are listed in Table S1.

### Immunocytochemistry

Cells were immunolabeled as described previously^6^. Cells were washed with PBS and then fixed with 4% paraformaldehyde in phosphate-buffered saline (PBS) for 15 min at room temperature. After rinsing with 0.1% Triton three times, Cells were blocked with blocking one (Nacalai Tesque) for 1 h, then incubated with primary antibodies overnight at 4 °C. After washing three times by PBS, cells were incubated with appropriate second antibodies and DAPI (Wako) for 2 h at room temperature. The primary antibodies and their working dilutions were as follows: mouse anti-Oct3/4 (1:1,600, BD Pharmingen, San Diego, CA), mouse anti-Mitf (1:100, Abcam, Cambridge, MA), rabbit anti-Pax6 (1:600, Covance, Munich, Germany), rabbit anti-Pax6 (1:500, Abcam), sheep anti-Chx10 (1:200, Exalpha Biologicals, Shirley, MA, USA), rabbit anti-ZO-1 (1:300, Thermo Fisher Scientific), mouse anti-Bestrophin (1:500, Abcam), and mouse anti-N-cadherin (1:200, Cell Signaling Technology, Danvers, MA, USA). The secondary antibodies used were as follows: anti-mouse IgG, anti-rabbit IgG, and anti-sheep IgG conjugated with Alexa488 or Alexa596 (1:1,000, Jackson Immunoresearch Laboratories Inc., West Grove, PA). For labeling of F-actin, fixed cells were treated with blocking one and then treated with Rhodamine-X-conjugated phalloidin (Wako) and DAPI for 2 h at room temperature. For lectin labeling for hiPSC, fixed cells were blocked with blocking one and then treated with FITC-labeled recombinant BC2L-C N-terminal domain (rBC2LCN-FITC, Wako) and DAPI for 2 h at room temperature. Labeled cells were imaged with a confocal laser-scanning microscope with GaAsP detectors (LSM800, Zeiss, Jena, Germany) using a 20× objective lens (NA 0.75, Zeiss) or 40× objective lens (NA 1.2, Zeiss).

To evaluate the purity of hiPSC-RPE, cells were treated with 0.25% Trypsin/EDTA for 15 min, dissociated into single cells by gentle pipetting, passed through a 35-μm cell strainer, and seeded on iMatrix 511-coated 3.5 cm culture dishes at a density of 1.0 × 10^5^ cells/cm^2^. Cells were cultured for 7–8 days in RPE growing medium until confluency. Then, the cells were fixed with 4% paraformaldehyde in PBS for 15 min at room temperature and subjected to immunostaining with anti-Chx10 and anti-Mitf antibodies. Labeled cells were imaged with a confocal laser-scanning microscope with GaAsP detectors. At least three visual fields were randomly selected. The number of PAX6-positive cells and MITF-positive cells were counted. Over 800 cells were counted in one sample. The cell purity was determined as the percentage of the number of positive cells to the number of total cells.

### Transepithelial electrical resistance (TER) measurement

hiPSC-RPE were seeded on the transwell insert for TER measurement using the electrical resistance system (Millicell, Millipore, Billerica, MA). A test electrode of the electrical resistance system was connected to the input port on the meter and confirmed at the value of 1,000 Ω. The electrode was rinsed with 70% ethanol for 15 min, and then with RPE maintenance medium for 15 min. Measurement was performed within 10 min after removal from the incubator to avoid reducing temperature. Net TER (Ω cm^2^) was calculated by subtracting the value of a blank insert from the experimental value and multiplying by the area of the insert membrane.

### Phagocytosis assay

hiPSC-RPE sheets were incubated in the RPE maintenance medium containing 7 μg of pH-Rhodo-labeled bioparticles (Thermo Fisher Scientific) at 37 °C in incubators for 4 h. As a negative control, hiPSC-RPE sheets were cultured in the presence of pH-Rhodo-labeled bioparticles at 4 °C. The cells were fixed with 4% paraformaldehyde in PBS, washed with PBS three times, and stained with phalloidin and DAPI. Fluorescent signals were imaged with a confocal laser-scanning microscope with GaAsP detectors using a 20× or 40× objective lens.

### Image processing and prediction model analysis

F-actin labeled microscopic images (total 54 images, 20× magnification) were processed by CL-Quant (Nikon corp., Tokyo, Japan) by designing filter sets according to the manufacture’s protocol. The labeled image processing and morphological feature measurement procedures are illustrated in Fig. S6. After cell recognition (Fig. S6A), cells with size < 500 pixels (305 μm^2^) were deleted from the recognized cell data because they tended to include mis-recognized objects or noise. After the data cleansing, 8 morphological features were measured by CL-Quant: (1) area, (2) compactness, (3) inner radius, (4) length, (5) length–width ratio, (6) outer radius, (7) perimeter, and (8) width (Table S2). The morphological features were carefully selected to eliminate multi-collinearity issues. After measuring cellular objects from each image, three replicate datasets consisting of 100–200 cells randomly picked from total cells by bootstrap in one image were produced from the original data. For 162 samples (= 54 images × 3), each average (AVE) and standard deviation (SD) of 100–200 cells were summarized and listed as explanatory variable sets (16 parameters) for each sample (Fig. S6B). The experimentally determined TER values (ranging from 70 to 390 Ω cm^2^) per each cellular sample were tagged as objective variables.

Before the construction of a prediction model, hierarchical clustering (average linkage) was applied to analyze the data distribution (Fig. S7A). The clustering indicated that the total data could be clustered in two groups: one cluster of which the majority correlated with high TER (group A, the right cluster) and the other cluster of which the majority correlated with low TER (group B, the left cluster). Although there was a general tendency of correlation between the morphological pattern and its functional TER value, there also existed a sub-population of samples that did not follow this pattern. In this analysis, we modeled the “primary morphological correlation”. Samples (> 250 TER) from group A and samples (< 150 TER) from group B were selected as “primary cells that represent the TER value”. The TER prediction model and TER-positive (> 250 TER) or negative (< 150 TER) discrimination model were constructed using the Least Absolute Shrinkage and Selection Operator regression as previously described^21^. The selected samples (total n = 98) were used for training the models. For checking their prediction performance of the models, the correlation of experimentally determined values (TER values, x-axis) and prediction values (predicted TER values only from the morphological features, y-axis) was plotted. The performance was also evaluated by root mean square error (RMSE) value, which indicates the error between the experimentally determined value and the predicted value. The accuracy, sensitivity, specificity, and its prediction probabilities were calculated for assessment of the discrimination performance. The performance was validated through leave-one-out cross-validation.

To adopt the constructed morphological TER prediction model to the non-labeled phase-contrast microscopic images, the 2 lots (6 samples), which showed high TER (mean TER value 342 ± 26 Ω cm^2^) and low TER (mean TER value 150 ± 12 Ω cm^2^) were selected for image processing and prediction (Figs. 5A and S8). From the non-labeled images, randomly chosen individual cells (~ 500 cells) were traced using the Cell Magic Wand tool (https://github.com/fitzlab/CellMagicWand) in ImageJ/Fiji with minimal manual assistance. By the traced clearer border of cells, the phase-contrast images were processed with the original filter set by CL-Quant as illustrated in Fig. S8A. From the recognized image, the morphologies of individual cells were measured by CL-Quant. From the measured group of cellular objects, their average and SD of 8 morphological features were measured to list 16 explanatory variables. These variables were applied to the constructed TER prediction model and TER-positive or -negative discrimination model, and their prediction performances were evaluated in the same manner.

To identify whether our model could be used to predict TER values of RPE sheets across cell lines, six different hiPSC-RPE sheets derived from three different cell lines were generated in a clinical manufacturing facility. Three RPE sheets were produced from the iRTA-01 cell line on different dates. Two RPE sheets were derived from the 253G1 hiPSC line, and one sheet was derived from the Ff-I01 hiPSC line. Phase-contrast images of the six hiPSC-RPE sheets were processed for morphological analysis as described above. The morphologies of individual cells were applied to the constructed TER prediction model, and their prediction performances were evaluated as described above.

Data processing and model analyses were coded with R 3.4.4. (https://cran.r-project.org). The codes used for the model construction are available at https://mega.nz/folder/zhA0SYAQ#g3uRzUiGKYbdDJmM6t3Lvw.

### Statistical analysis

Data in the experimental sections are expressed as means ± SEM. All sets of experiments were performed at least three times. The statistical significance of the differences between groups was determined using unpaired *t*-tests with GraphPad Prism 7 (GraphPad Software, Inc., San Diego, CA). Probability values less than 5% were considered statistically significant.

## Supplementary information

Supplementary Legends.

Supplementary Figure S1.

Supplementary Figure S2.

Supplementary Figure S3.

Supplementary Figure S4.

Supplementary Figure S5.

Supplementary Figure S6.

Supplementary Figure S7.

Supplementary Figure S8.

Supplementary Figure S9.

Supplementary Figure S10.

Supplementary Table S1.

Supplementary Table S2.

Supplementary Table S3.

Supplementary Table S4.
