# Supplementary material for: Reproducible production and image-based quality evaluation of retinal pigment epithelium sheets from human induced pluripotent stem cells
Source: Sci Rep. 2020 Sep 1;10:14387. doi: 10.1038/s41598-020-70979-y (PMC7462996; doi:10.1038/s41598-020-70979-y)
Supplement: Supplementary file 1 — Supplementary Legends. [file 41598_2020_70979_MOESM1_ESM.docx]

# Supplementary figure legends

## Supplementary Figure 1

## Human iPSC 1383D6 exhibited pluripotent features under feeder-free culture conditions

**(A)** Low magnification phase-contrast images showing the morphology of 1383D6 hiPSC colonies in 4 days culture.

**(B)** High magnification phase-contrast images phase-contrast images showing the large nuclei and clear cytoplasms of 1383D6 hiPSC colonies in 4 days culture.

**(C)** Representative immunostaining for the pluripotency marker OCT4 in 1383D6 hiPSC colonies.

**(D)** Representative rBC2LCN staining on 1383D6 hiPSC colonies.

**(E)** Representative immunostaining for the pluripotency marker OCT4 in 1383D2 hiPSC colonies.

**(F)** Representative immunostaining for the pluripotency marker OCT4 in A18945 hiPSC colonies. Scale bars: 250 μm (A) and 100 μm (B, C, D, E)

## Supplementary Figure 2

## NIC treatment promoted the differentiation of RPE cells.

**(A)** Representative photomicrographs of MITF-positive cells on differentiation day 24. The majority of induced cells were positive for MITF on day 24. Note that NIC treatment decreased the area of MITF-negative cells (shown with dotted lines).

**(B)** Representative photomicrographs of immunostaining for MITF and PAX6. hiPSC were differentiated in the presence or absence of NIC, fixed on day 24, and then subjected to immunostaining. Note that NIC treatment decreased cells that were double-positive for MITF and PAX6.

**(C, D, E)** NIC treatment promoted the differentiation and maturation of RPE progenitors to RPE. The percentages of MITF-positive, PAX6-positive cells (C) and the percentage of MITF-positive, PAX6-negative cells (D) were determined using immunostaining on day 12, 18, and 24. The ratios of of MITF-positive, PAX6-negative cells to MITF-positive cells was determined using immunostaining for PAX6 and MITF on day 24 and expressed as percentages (E).*** *P* < 0.001, compared to control.

Scale bars: 50 μm (A, B)

## Supplementary Figure 3

## High reproducibility of the RPE6iN method across different trials and cell lines

**(A)** MITF-positive cells were reproducibly induced using the RPE6iN method on day 35 in three independent sets of experiments. A set of experiments was composed of three wells containing RPE6iN-treated hiPSC. We independently performed the set of experiments three times. Each column in the figure represents data from a set of experiments. Each dot corresponds to a well in a set of experiments. MITF-positive cells were quantified using immunostaining. Note that every trial showed similar MITF induction efficiency.

**(B)** Pigmented areas of 6 wells plates were reproducibly induced using the RPE6iN method on day 35 in three independent sets of experiments. A set of experiments was composed of three wells contianing RPE6iN-treated hiPSC. We independently performed the set of experiments three times. Each column in the figure represents data from a set of experiments. Each dot corresponds to a well in a set of experiments. Pigmented areas were quantified using ImageJ. Note that each trial consistently demonstrated similar pigment induction efficiency.

**(C)** Differentiation of RPE progenitors from 1383D2 using RPE6iN. Representative immunostaining for MITF and PAX6 on day 12. Clone 1383D2 hiPSC were differentiated using the RPE6iN protocol, fixed on day 12, and then subjected to immunostaining.

**(D)** Differentiation of RPE progenitors from A18945 using RPE6iN. Representative immunostaining for MITF and PAX6 on day 12. Clone A18945 hiPSC were differentiated using RPE6iN, fixed on day 12, and then subjected to immunostaining.

Scale bars: 50 μm (C, D)

## Supplementary Figure 4

**Phagocytosis ability of hiPSC-RPE sheets in 4 weeks.**

When these bioparticles are phagocytized by RPE, they become fluorescent inside the RPE in response to pH changes. Representative images of hiPSC-RPE sheets after 4 hours of exposure to pH-Rhodo-labeled bioparticles (green) at 4°C (negative control) or 37°C. Nuclei were counterstained with DAPI (blue). Scale bar, 20 μm.

## Supplementary Figure 5

**Abnormal F-actin architecture in hiPSC-RPE sheets with low TER values**

**(A)** Normal localization of the tight junction protein ZO-1 in RPE cells. RPE cells were processed for immunostaining. The x-z confocal section (lower panel) showed that ZO-1 was localized to the apical surface of cells.

**(B)** Normal localization of N-cadherin and F-actin in RPE cells. RPE cells were processed for immunostaining. x-z confocal sections (lower panel) showed that N-cadherin co-localized with F-actin in the lateral membrane of RPE cells.

**(C)** Representative photomicrographs of ZO-1 and F-actin staining of RPE sheets with low TER values (126 Ω⋅cm^2^) in x-y and x-z confocal sections. HiPSC-derived RPE cells were cultured in transwells and subjected to TER measurement and immunostaining. Arrowheads indicate stacked RPE cells with mislocalized expression of ZO-1 and F-actin.

**(D)** Phalloidin staining images of representative x-z sections of RPE sheets with high TER values (344 Ω⋅cm^2^) and those with low TER values (129 Ω⋅cm^2^). Magenta and green represent F-actin and nuclei, respectively. Note that lateral F-actin was lost in RPE sheets with low TER values (arrowheads).

Scale bars: 10 μm (A, B, D) and 20 μm (C)

## Supplementary Figure 6

## Schematic procedure of image processing and data processing of F-actin-labeled images

**(A)** The procedure of image processing and cell recognition in F-actin-labeled images.

**(B)** The procedure of data processing and hierarchical clustering of morphological features obtained from F-actin-labeled images.

## Supplementary Figure 7

## Hierarchical clustering of morphological features and construction of a machine learning-based prediction model

**(A)** Hierarchical clustering of morphological features obtained from F-actin-labeled images of hiPSC-RPE sheets. Eight morphological features, with corresponding average and SD for RPE sheets with different TER, values were categorized by hierarchical clustering. The majority of Group A was correlated with high TER values while the majority of Group B was correlated with low TER values.

**(B)** The concept of model construction by machine learning. HiPSC-RPE sheets have complex morphological features causing biological heterogeneity. Following the reduction of noise in the analysis and extraction of the majority of information among RPE sheets with low TER values and high TER values, the prediction model was constructed based on major trends in morphological features.

## Supplementary Figure 8

## Schematic procedure of image processing and TER prediction of phase-contrast images

**(A)** The procedure for image processing and cell recognition in label-free, phase-contrast images.

**(B)** The procedure for prediction of phase-contrast images by the prediction model constructed from F-actin-labeled images.

## Supplementary Figure 9

**TER prediction of six hiPSC-RPE sheets generated from three different hiPSC lines in a clinical manufacturing facility**

RPE sheets were produced from various hiPSC lines (Ff-I01, iRTA, and 253G1) using different differentiation protocols in a clinical manufacturing facility. The morphological features of six RPE sheets derived from the different lines were extracted and subjected to our prediction model generated from the hiPSC line 1383D6. Results indicate that our prediction method can be applied across multiple cell lines and other facilities.

## Supplementary Figure 10

**Cryopreservation of hiPSC-derived RPE cells for a hiPSC-RPE cell bank**

1. Timetable for cryopreservation of hiPSC-derived RPE cells.

Upper: HiPSC-RPE cells were frozen on day 49 of the RPE6iN method in Stem-Cell-Banker, a DMSO-free, GMP grade cryopreservation medium.

Lower: Frozen hiPSC-RPE cells were thawed and cultured on iMatrix-coated dishes for 14 days.

**(B)** Phase-contrast image of hiPSC-derived RPE cells derived from frozen stocks.

Frozen stocks of hiPSC-RPE cells can be recovered after thawing and culturing for 5 days.

**(C)** Phase**-**contrast image of hiPSC-derived RPE cells with polygonal shapes.

Thawed hiPSC-derived RPE cells showed a typical RPE morphology after culture for 14 days.

Scale bars: 100 μm (A, B)

# Supplementary tables

**Supplementary Table 1: List of primers used for qPCR analysis**

## Supplementary Table 2: Definitions of basic morphological features used for cell measurement

The average and standard deviation of the eight morphological features measured using RPE images were used for hierarchical clustering and prediction modeling: area, inner radius, outer radius, length, width, length-width ratio, perimeter, and compactness

## Supplementary Table 3: Parameters and weights affecting the TER prediction model.

“Average of length-width ratio” and “SD of compactness” had negative effects in the TER prediction model, whereas “Average of width” and “SD of length/width ratio” had positive effects

## Supplementary Table 4: Parameters and weights affecting the TER discrimination model

“SD of compactness” had the strongest negative effect in the discrimination model, whereas “Average of width” had the strongest positive effect.
