# Supplementary figures and images for: Reproducible production and image-based quality evaluation of retinal pigment epithelium sheets from human induced pluripotent stem cells
Source: Sci Rep. 2020 Sep 1;10:14387. doi: 10.1038/s41598-020-70979-y (PMC7462996; doi:10.1038/s41598-020-70979-y)

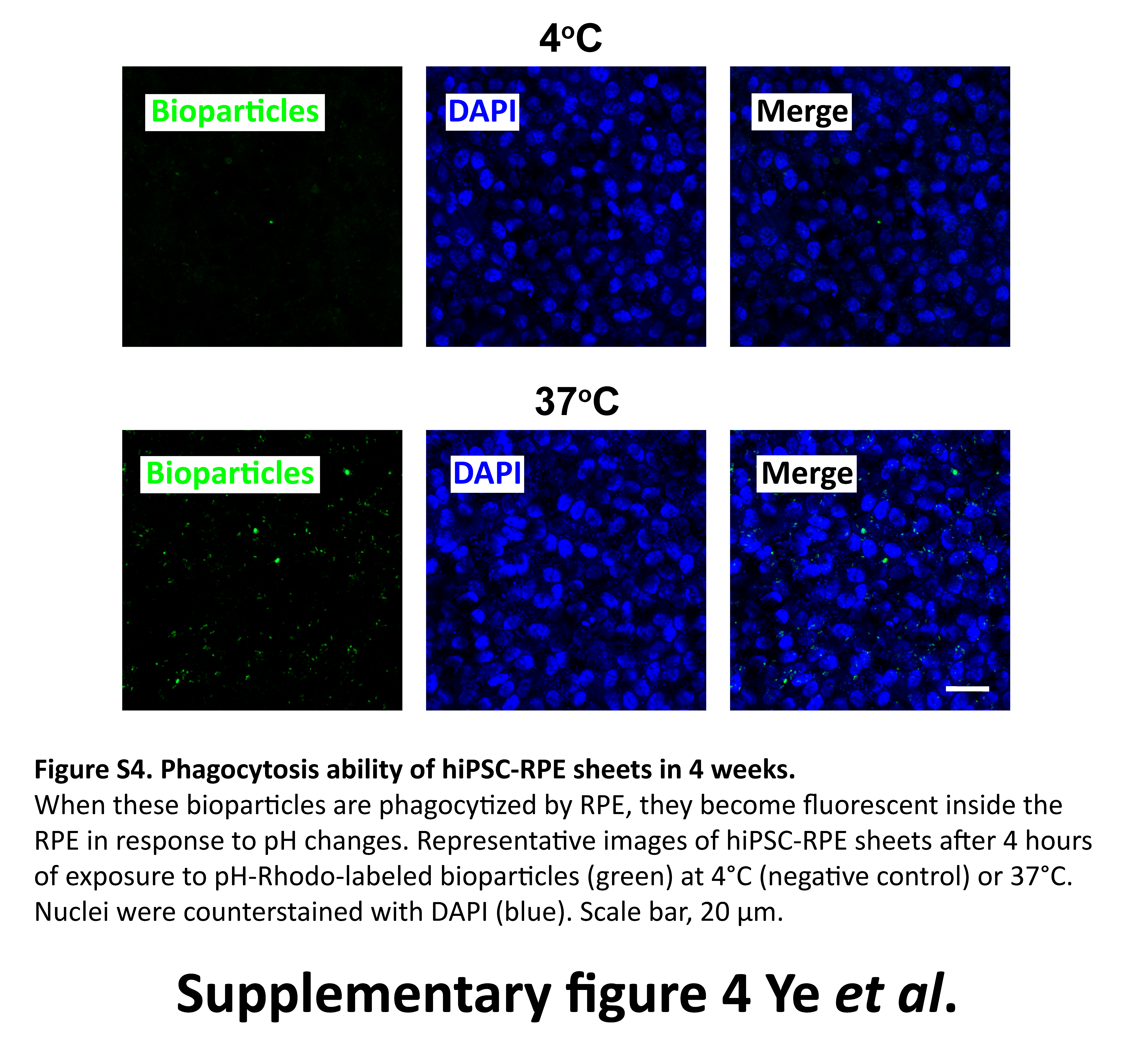

Supplement: Supplementary file 5 — Supplementary Figure S4. [file 41598_2020_70979_MOESM5_ESM.png]

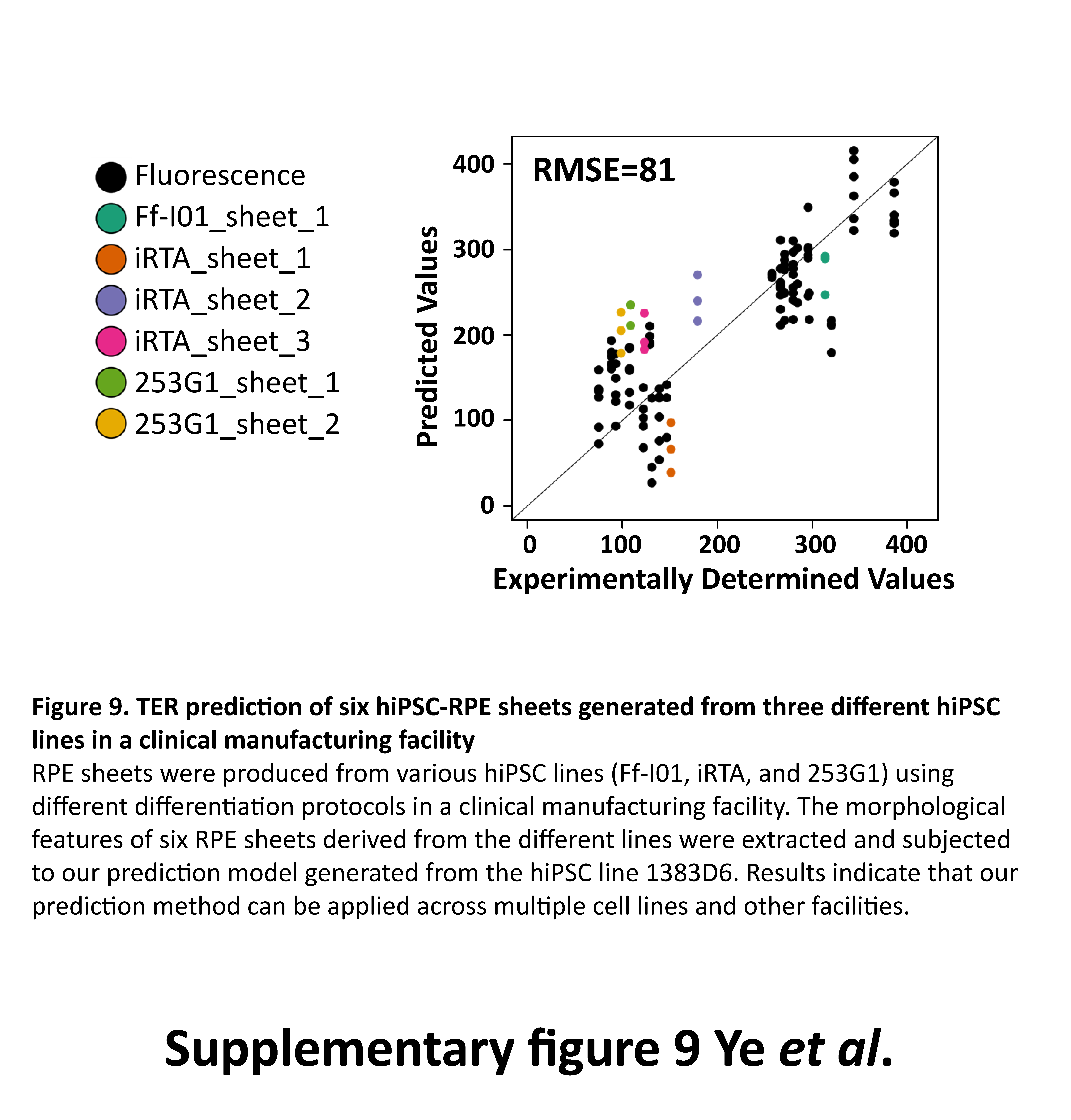

Supplement: Supplementary file 10 — Supplementary Figure S9. [file 41598_2020_70979_MOESM10_ESM.png]

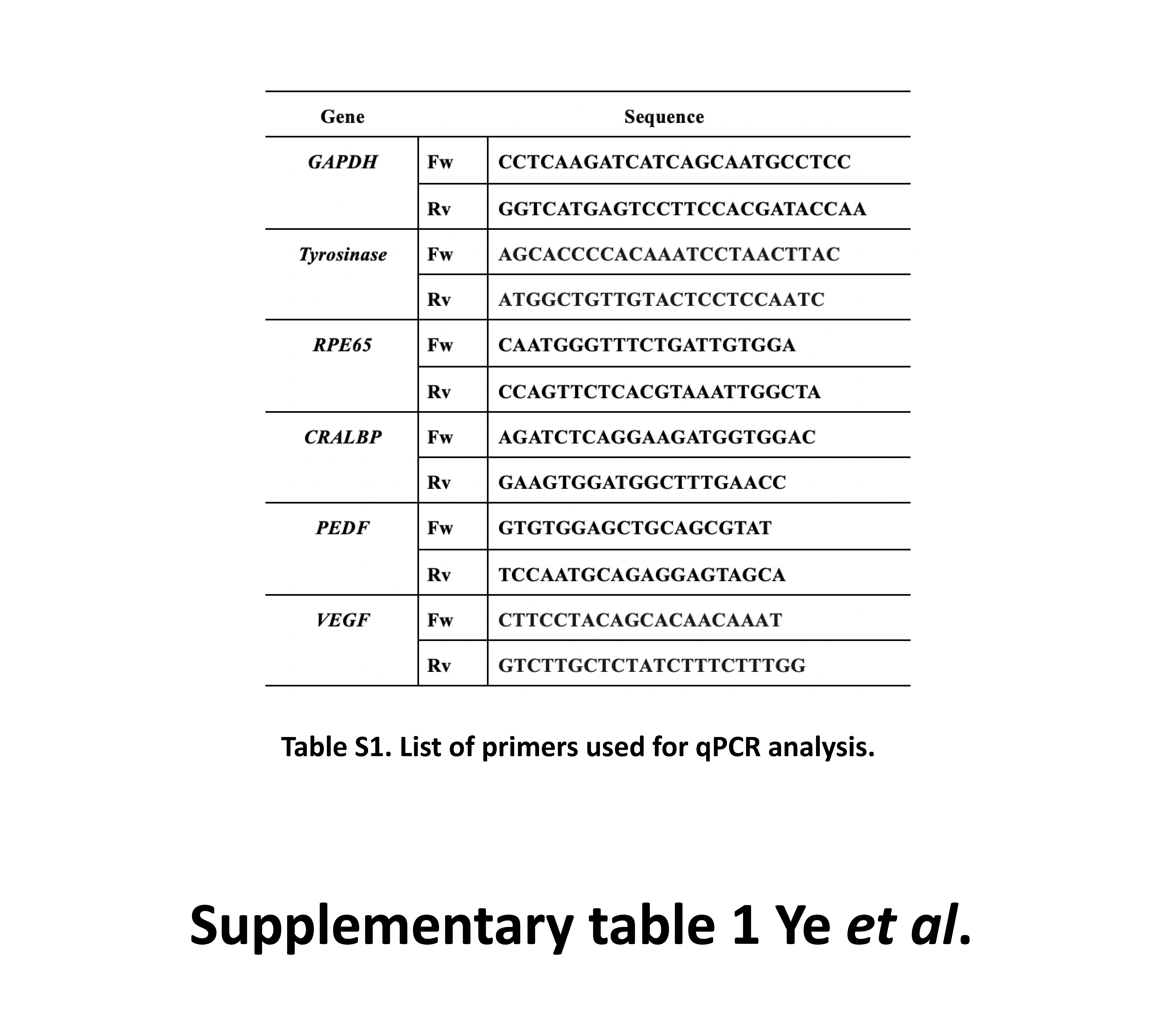

Supplement: Supplementary file 12 — Supplementary Table S1. [file 41598_2020_70979_MOESM12_ESM.png]

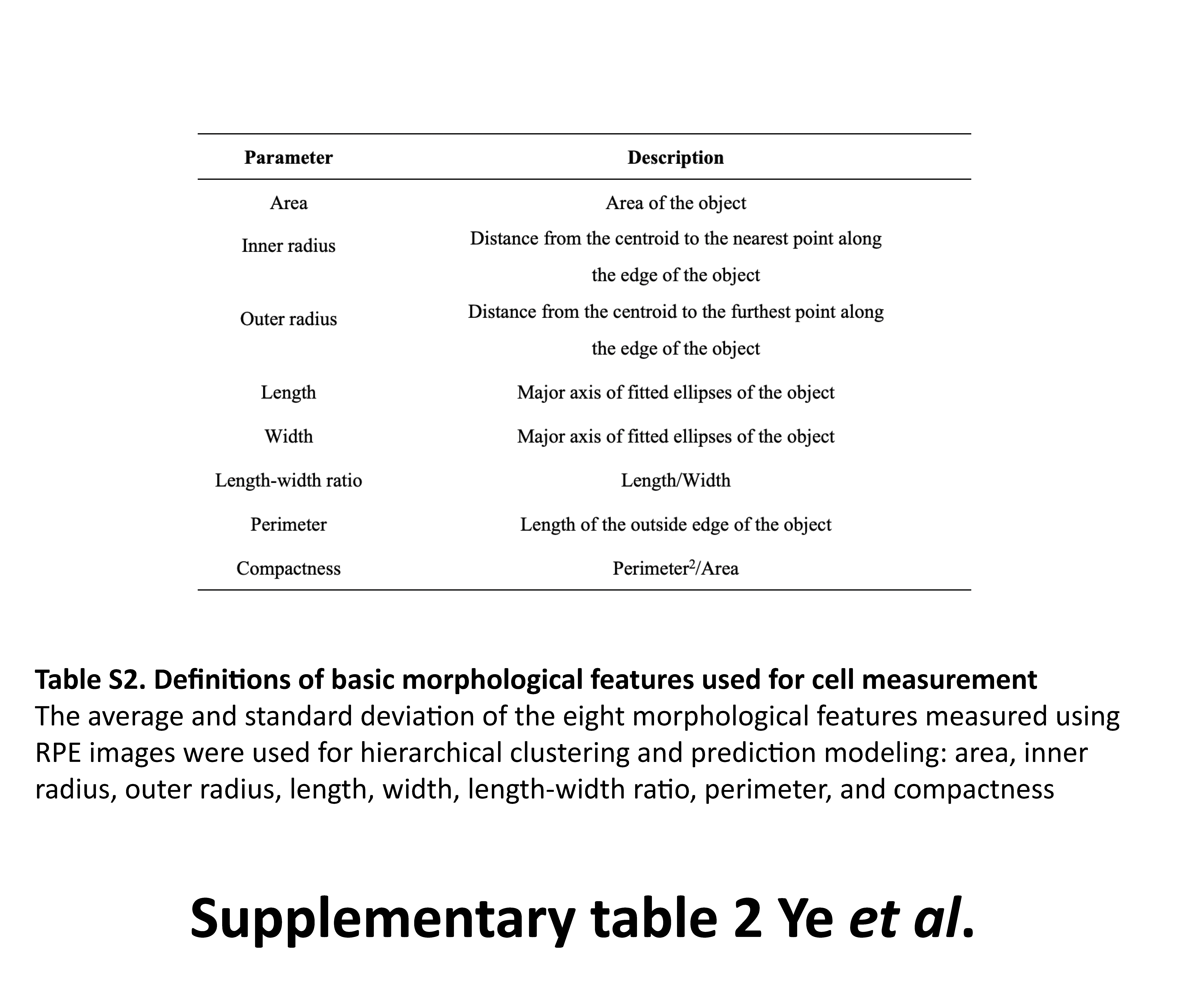

Supplement: Supplementary file 13 — Supplementary Table S2. [file 41598_2020_70979_MOESM13_ESM.png]

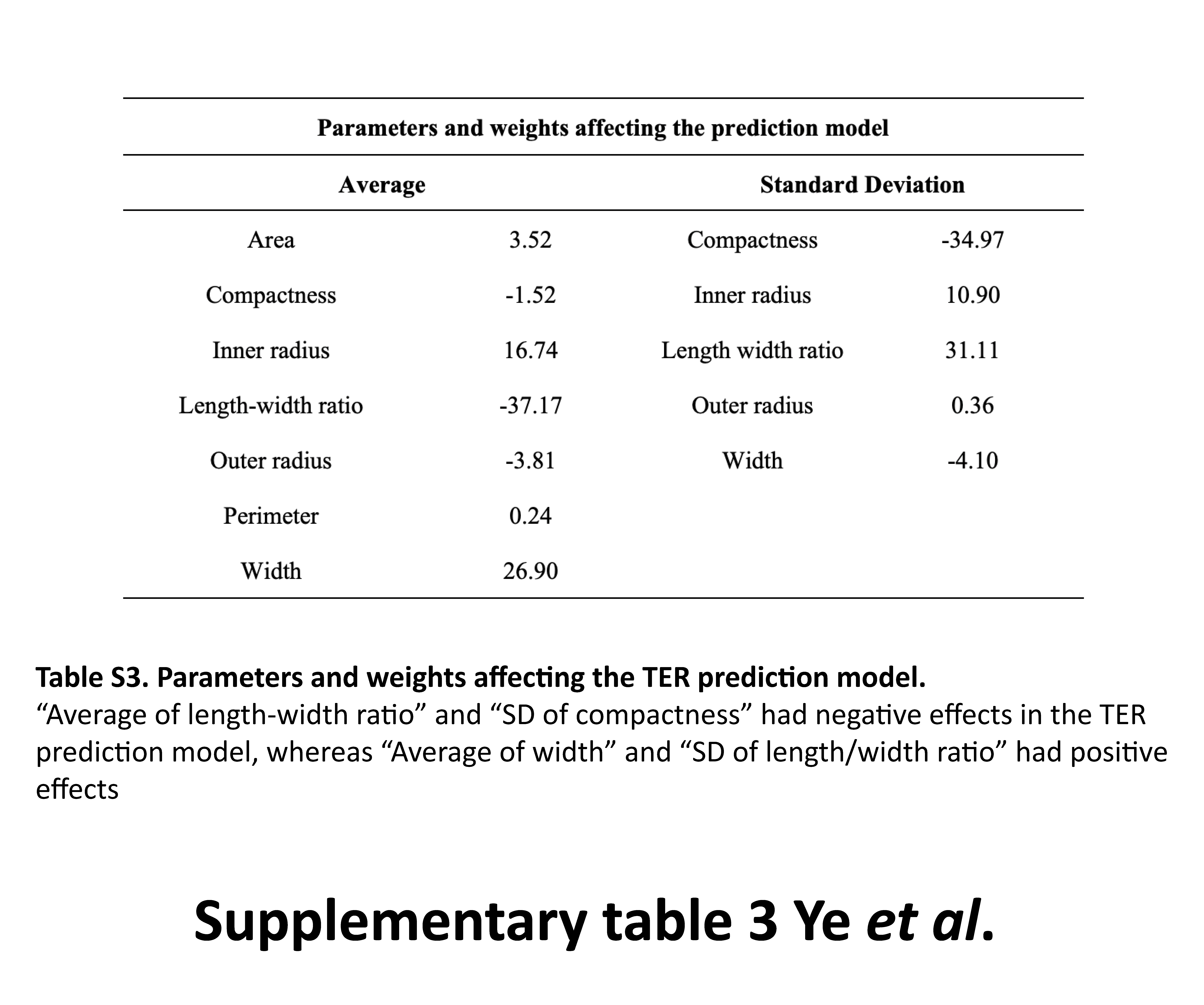

Supplement: Supplementary file 14 — Supplementary Table S3. [file 41598_2020_70979_MOESM14_ESM.png]

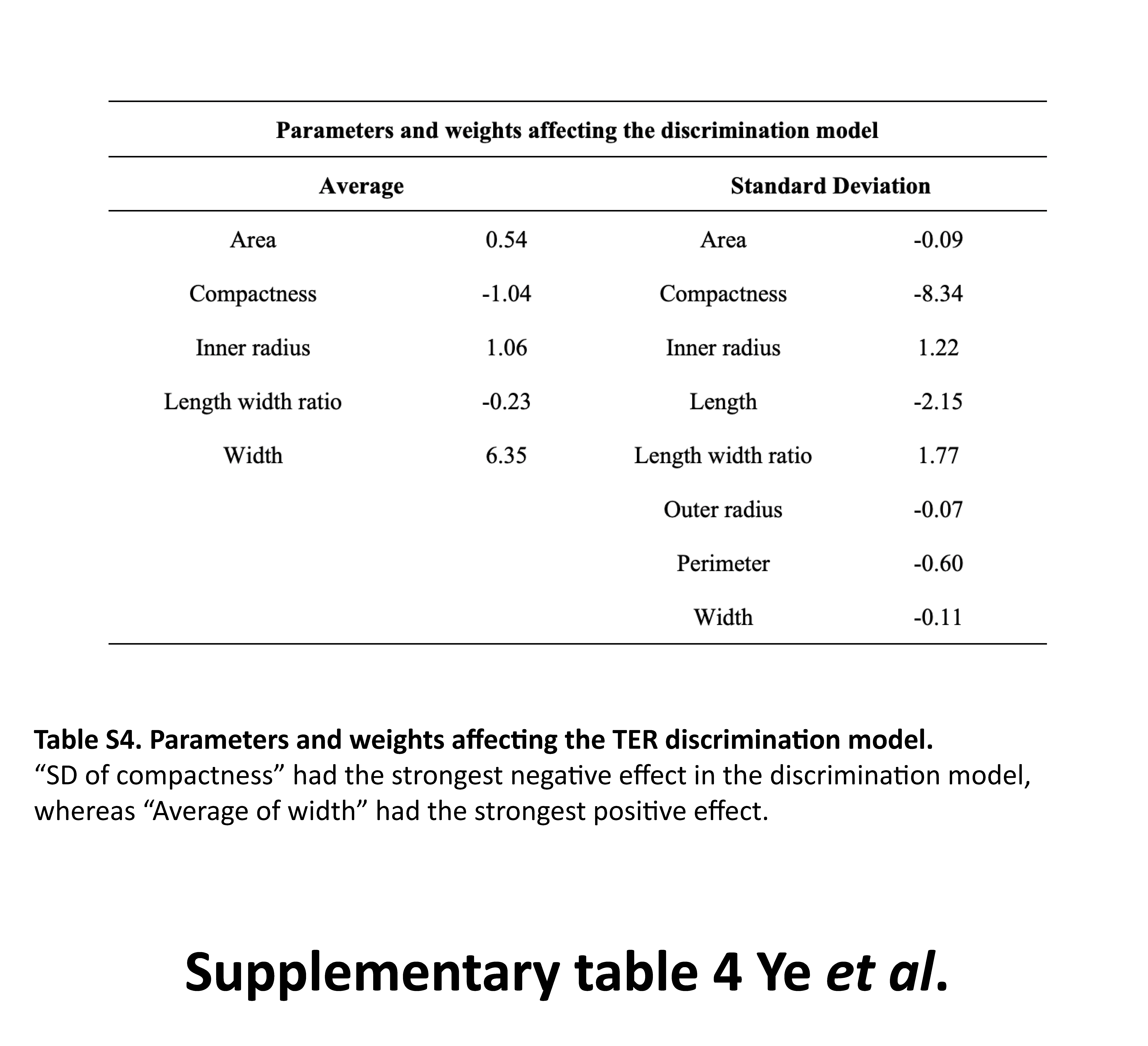

Supplement: Supplementary file 15 — Supplementary Table S4. [file 41598_2020_70979_MOESM15_ESM.png]
